# Characterization of the SOS meta-regulon in the human gut microbiome

**DOI:** 10.1093/bioinformatics/btt753

**Published:** 2014-01-08

**Authors:** Joseph P. Cornish, Neus Sanchez-Alberola, Patrick K. O’Neill, Ronald O'Keefe, Jameel Gheba, Ivan Erill

**Affiliations:** Department of Biological Sciences, University of Maryland Baltimore County (UMBC), Baltimore, MD 21250, USA

## Abstract

**Motivation:** Data from metagenomics projects remain largely untapped for the analysis of transcriptional regulatory networks. Here, we provide proof-of-concept that metagenomic data can be effectively leveraged to analyze regulatory networks by characterizing the SOS meta-regulon in the human gut microbiome.

**Results:** We combine well-established *in silico* and *in vitro* techniques to mine the human gut microbiome data and determine the relative composition of the SOS network in a natural setting. Our analysis highlights the importance of translesion synthesis as a primary function of the SOS response. We predict the association of this network with three novel protein clusters involved in cell wall biogenesis, chromosome partitioning and restriction modification, and we confirm binding of the SOS response transcriptional repressor to sites in the promoter of a cell wall biogenesis enzyme, a phage integrase and a death-on-curing protein. We discuss the implications of these findings and the potential for this approach for metagenome analysis.

**Contact:** erill@umbc.edu

**Supplementary information:** Supplementary data are available at *Bioinformatics* online.

## 1 INTRODUCTION

The unprecedented coverage provided by next-generation sequencing technologies has enabled direct sequencing of heterogeneous bacterial populations in their natural habitat. Metagenomics projects, therefore, offer a unique window into the genetic composition of microbial ecosystems (Riesenfeld *et al.*, 2004). To date, a substantial amount of work has focused on pathway analysis as a method to analyze and compare metagenomic populations (De Filippo *et al.*, 2012). This approach is particularly suited for reconstructing the multispecies genetic organization of metabolic pathways across a metagenome (Abubucker *et al.*, 2012; Nelson *et al.*, 2010). In contrast, non-metabolic transcriptional regulatory networks (TRNs), such as stress responses, present a different challenge. These networks are typically defined by the functional regulation of their components, rather than by their biochemical interconnections, and their genetic composition can vary significantly across bacteria (Cornish *et al.*, 2012; Erill *et al.*, 2007; Lozada-Chavez *et al.*, 2008; Rodionov *et al.*, 2005). Hence, the mere presence of a particular gene or set of genes within a metagenomic sample does not imply their belonging to a given TRN or the existence of such a network in the sample. Membership to a TRN can be instead defined by the ability of a regulating transcription factor to bind upstream of and directly regulate an operon (Ptashne and Alexander, 2002). In well-studied bacterial transcription factors, the binding motif is known to be conserved at the class or phylum levels (Gorbunov *et al.*, 2010; Kotelnikova *et al.*, 2005; McGuire *et al.*, 2000). If the binding motif for the regulating transcription factor is known and can be shown to be conserved for a given subset of the sampled taxa, it is hence theoretically possible to reconstruct its associated composite TRN, or meta-network, at the metagenome level by analyzing sequence data from metagenomic projects. Conservation of the binding motif can be assessed through comparative genomics analyses, which can also be used to validate robust multispecies scoring matrices that are well suited for metagenomic analyses (Cornish *et al.*, 2012). The analysis of regulatory networks at the metagenome level presents several potential advantages over genomic analyses. First, it leverages a large and increasing amount of data for regulatory network analysis. Second, it complements a genome-centered perspective by providing a glimpse of the regulatory network organization in its natural environment. Finally, it can capture the contribution of uncultured organisms and mobile genetic elements that are poorly represented in genomic data. Here, we explore this approach by analyzing the composition of the bacterial SOS regulon in the human gut microbiome through *in silico* and *in vitro* analyses, providing proof-of-concept for the use of metagenomic data in the inference of regulatory meta-networks.

The SOS response presents several properties that make it a suitable target to validate this type of analysis. It is a well-studied, widespread and relatively large network (∼40 genes in *Escherichia coli*) aimed primarily at addressing DNA damage through the activation of repair enzymes, translesion synthesis polymerases and cell division inhibitors (Fernandez De Henestrosa *et al.*, 2000; Wade *et al.*, 2005). It is also of clinical relevance due to its implication in virulence, mutagenesis and dissemination of antibiotic resistance through the activation of mobile genetic elements (Abella *et al.*, 2007; Kelley, 2006; Wade *et al.*, 2005). In addition, it is governed by a repressor (LexA) with a remarkably different distribution in the two clades that constitute Bacteroidetes, but it targets a well-defined binding motif in the Firmicutes (Erill *et al.*, 2007; Segata *et al.*, 2012).

## 2 METHODS

### 2.1 Metagenome and genome data

Metagenome data were obtained from the MetaHit project (Qin *et al.*, 2010). Only records from 86 healthy patients were used for analysis. A library of 122 gut microbiome complete genomes was compiled as reference for BLAST analysis and subsequent taxonomic typing (Qin *et al.*, 2010).

### 2.2 Identification of putative LexA-binding sites

A multispecies collection of experimentally validated Gram-positive LexA-binding sites and a reference set of LexA-regulated clusters of orthologous genes (COGs) across the bacteria domain were derived from the published literature (Supplementary Tables S1 and S2). Putative LexA-binding sites were located by scoring all metagenome sequences on both strands using the *R_i_* index as implemented in FITOM (Erill and O’Neill, 2009; Schneider, 1997). Only putative sites with scores >1.5 standard deviations below the mean of the original collection (>8.36 bits) were considered for further analysis. Promoter regions were defined as spanning from −300 to +50 bp of the translational start site of a predicted protein-coding gene (McGuire *et al.*, 2000; Rodionov *et al.*, 2004; Sanchez-Alberola *et al.*, 2012). Predicted protein-coding genes in the same orientation and with an intergenic distance <100 bp were considered to constitute an operon. All identified LexA-binding sites were associated with the predicted protein-coding gene in the metagenome sequence whose translational start site was closest to the LexA-binding site.

### 2.3 Functional annotation and taxonomic filtering

Predicted protein-coding genes and their COG annotations were obtained from the MetaHit gene dataset (Qin *et al.*, 2010). Protein-coding genes were functionally annotated using BLASTP against a database of 122 gut microbiome complete genomes with an *E*-value threshold of 10^−^^10^ on the UMBC High Performance Computing Facility. The predicted protein function and full taxonomic information for the best BLAST hit associated with each gene was obtained from the NCBI Protein and Taxonomy databases, respectively. Phylum-level taxonomic information was used to filter identified sites by excluding all sites not associated with protein-coding genes assigned to the Firmicutes or the Actinobacteria. Site and gene data were integrated in a mySQL database and interrogated with custom Structured Query Language (SQL) queries. 

### 2.4 Identification of prototypical SOS COGs

For all COGs reported in the MetaHit data, the cumulative distribution of site *R_i_* scores was computed by counting the number of filtered putative LexA-binding sites (Supplementary Table S3) above a given score threshold, using 1 bit intervals in the 12–20 bit score range. Prototypical SOS COGs were defined as those having a regression coefficient of determination (*R^2^*) >0.85 in the 12–20 bit range, at least one site with score above the mean (16 bits) and a minimum of 10 sites in the 12–20 bit range. The *R^2^* cutoff value of 0.85 was set simply by ranking COGs according to *R^2^* value and gradually decreasing the *R^2^* threshold until the inclusion of additional COGs corresponding to known SOS genes (Supplementary Table S2) incurred the inclusion of a larger number of COGs not known to be associated with SOS genes.

### 2.5 *In vitro* validation of LexA-binding sites

The *Bacillus subtilis lexA* gene was overexpressed in a pET15b vector provided by Dr Jordi Barbé. The *B.subtilis* LexA protein was purified as described previously (Guerin *et al.*, 2009). DNA probes were constructed using two complementary 61-bp synthetic oligonucleotides (Supplementary Table S4). Electrophoretic mobility shift assays (EMSA) experiments were performed as reported previously (Sanchez-Alberola *et al.*, 2012), using 40 nM of *B.subtilis* LexA and 20 ng of each Digoxigenin-marked DNA probe in the binding mixture. For EMSA competitive assays (Supplementary Fig. S1), 200-fold of either specific or non-specific non-labeled DNA was added to the binding mixture. In all cases, samples were loaded in 6% non-denaturing Tris-glycine poly-acrylamide gel. Digoxigenin-labeled DNA-protein complexes were detected following the manufacturer's protocol (Roche).

## 3 RESULTS AND DISCUSSION

### 3.1 Identification of LexA-binding sites in human gut microbiome metagenomic data

**Fig. 1.**
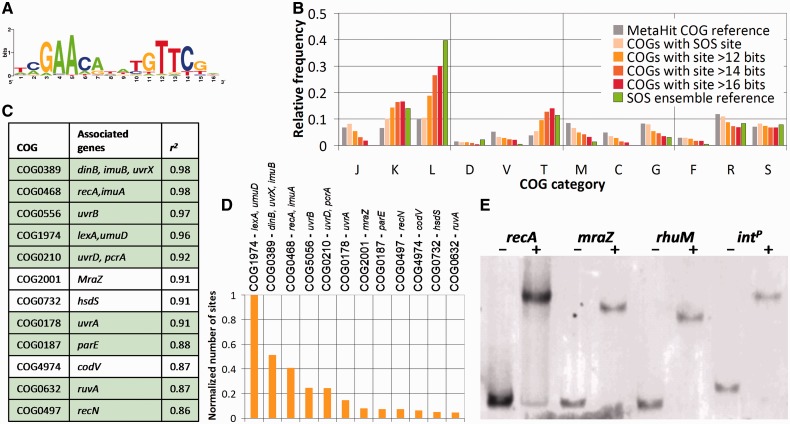
(**A**) Sequence logo for the interspecies LexA-binding motif used to search the MetaHit metagenome. (**B**) Distribution of representative COG categories as a function of score threshold and comparison with a reference distribution of known SOS COG categories. COG category abbreviations: C—energy production and conversion, D—cell cycle control and mitosis, F—nucleotide metabolism and transport, G—carbohydrate metabolism and transport, J—translation, K—transcription, L—replication and repair, M—cell wall/membrane/envelop biogenesis, V—defense mechanisms, T—signal transduction, R—general functional prediction only, S—function unknown. Supplementary Fig. S3 shows the distribution for all COG categories. (**C**) List of top scoring SOS-related COGs as a function of the linear regression coefficient of determination (*R^2^*) for the site score cumulative distribution in the 12–20 bit range. Rows corresponding to known SOS genes (Supplementary Table S2) are shaded. (**D**) Normalized number of sites per putative SOS COG. The number of sites associated with each COG was normalized to the number of sites for COG1974. (**E**) EMSA of selected LexA-binding sites using *B.subtilis*-purified LexA protein. All experiments show two gel lanes, corresponding to the absence (−) and presence (+) of LexA protein. The *B.subtilis recA* promoter (leftmost experiment) was used as a positive control

We used a position-specific scoring matrix derived from a multispecies Gram-positive bacteria motifs (Fig. 1A; Supplementary Table S1) to search the combined 7.1 Gbp sequence of the human gut metagenome of 86 healthy individuals (Qin *et al.*, 2010; Cornish *et al.*, 2012). This led to the identification of more than half a million putative LexA-binding sites. As expected, average site scores were significantly higher in putative promoter regions (Supplementary Fig. S2). We filtered the initial results by requiring that putative LexA-binding sites be located in such upstream regions (−350 to +50 of predicted translational start site) and that their associated genes mapped to Gram-positive bacteria. This two-pronged filtering step resulted in a final count of >40 000 sites associated with >38 000 genes with products assigned to COGs (Supplementary Table S3). We validated this approach by analyzing the distribution of gene counts per COG category as a function of the score threshold (Fig. 1B; Supplementary Fig. S3) and comparing it with a reference ensemble of 18 known SOS regulons (Supplementary Table S2). The fit of the inferred network with the reference ensemble improves with increasing score threshold, leading to a progressive enrichment of all canonical SOS categories [repair/replication (L), signal transduction (T) and transcription (K)] except for cell cycle control (D). This canonical SOS category (Supplementary Table S5) is not enriched because SOS-regulated cell division inhibitors in Gram-positive bacteria were described after the creation of the COG database and their associated COGs are assigned to several different categories (Chauhan *et al.*, 2006; Kawai *et al.*, 2003; Ogino *et al.*, 2008; Tatusov *et al.*, 2000).

### 3.2 Functional analysis of the prototypical human gut SOS regulon

An interesting property of the use of metagenomic data to analyze TRNs is that it can provide an estimate of the relative gene composition of the TRN in its natural environment. We analyzed the gene composition of the human gut SOS meta-regulon contributed by Gram-positive bacteria by assessing the relative number of site counts per COG as a function of site score. As expected, COGs mapping to known SOS genes are enriched in high-scoring sites, capturing roughly half of the sites with scores >16 bits (∼200) in promoter regions. This trend is not seen outside promoter regions, where the percentage of high-scoring sites (>16 bits) for COGs mapping to known SOS genes is similar to that observed for all COGs (∼0.5%). To accurately identify prototypical SOS COGs, we first analyzed LexA-binding sites upstream of canonical SOS genes (*lexA* and *recA*) drawn from genomic sequences. For these reference genes, LexA-binding site scores follow a normal distribution with a mean of ∼16.2 bits (Supplementary Fig. S4). In its central range (12–20 bits), the cumulative distribution can be fit by a linear model, providing a simple means to define prototypical SOS COGs on the MetaHit data. Data for other well-known transcriptional regulators suggest that this is a general property of TF-binding site distributions upstream of regulated genes (Supplementary Fig. S5). We used the coefficient of determination (*R^2^*) of the linear regression for the cumulative distribution of site scores on each COG, together with the presence of at least one high-scoring site, as an indicator of the likelihood of a COG being a prototypical member of the SOS regulon. Using these criteria and a conservative cutoff, we identified 12 putative SOS COGs (Fig. 1C). Nine of these COGs correspond to well-established SOS genes, such as *lexA* and *umuD* (COG1974) or the excision repair helicase *uvrB* (COG0556). The remaining three are promising candidate additions to the SOS regulatory network. COG0732 maps to HsdS, a type I restriction endonuclease linked to virulence in *Streptococcus suis* (Li *et al.*, 2010). COG2001 is defined by the cell division protein MraZ, known to be involved in cell wall biogenesis and induced by antibiotic stress in *Pseudomonas aeruginosa* (Bagge *et al.*, 2004), and COG4974 maps to the *B.subtilis* CodV protein, a site-specific recombinase involved in chromosome partitioning (Sciochetti *et al.*, 2001). By relaxing the *R^2^*-cutoff, we identified several additional COGs linking the SOS network to cell wall biosynthesis and export processes potentially involved in virulence (Supplementary Table S6). COG0399, for instance, is linked with the biosynthesis of a phase-variable O antigen and the extracellular matrix of *Vibrio cholerae* (Rashid *et al.*, 2003; Seed *et al.*, 2012), whereas COG1136 is involved in antimicrobial peptide transport systems that have been linked to virulence in multiple species (Matthysse *et al.*, 1996; Pethe *et al.*, 2004; Pietiäinen *et al.*, 2009). The number of sites associated with each COG (Fig. 1D) reveals that the composite gut microbiome SOS regulon is dominated by four molecular functions: transcriptional repression (*lexA*), sensing of DNA-damage and stabilization of DNA (*recA*), translesion synthesis (*umuD*, *dinB*, *uvrX* and *imuB*) and excision repair (*uvrB*, *uvrD* and *uvrA*). In agreement with the experimental evidence uncovered in different phyla (Erill *et al.*, 2007), the comparatively large number of sites associated with error-prone polymerases highlights the importance of translesion synthesis as a primary component of the SOS response, aimed at guaranteeing the completion of chromosome replication in response to DNA damage. Previous work has analyzed the composition of the SOS response in Gram-positive bacteria through comparative genomics, recognizing cell division inhibition, but not translesion synthesis, as a key component of this system (Cornish *et al.*, 2012). Furthermore, none of the additional SOS COGs reported here was identified in the comparative genomics analysis. This illustrates how the environment-specific enrichment of functional groups in metagenomics data can enhance regulon analysis, complementing conventional analyses with population-based estimates of functional importance.

### 3.3 *In vitro* validation of selected binding sites

A further asset of leveraging metagenomics for TRN analysis is the ability to identify regulatory targets in uncultured organisms and mobile genetic elements that may be underrepresented in genomic data. We selected three high-scoring putative LexA-binding sites for *in vitro* analysis to confirm *in silico* results and to explore putative novel additions to the SOS network: an MraZ homolog (*mraZ*), a phage integrase (*int^P^*) and a death-on-curing protein (*rhuM*) (Supplementary Table S4). We validated binding to these sites through EMSA using purified *B.subtilis* LexA protein (Fig. 1E; Supplementary Fig. S1). Our results confirm that these LexA-binding sites are bound by LexA *in vitro*, suggesting that they are functional sites *in vivo* regulating the activity of their associated genes. The potential regulation of the cell division protein MraZ by the SOS response coincides with recent reports of distinct cell division regulators under control of LexA in Gram-positive bacteria and gives credence to the idea that the link between the SOS response and control of cell division may have evolved multiple times in this clade (Cornish *et al.*, 2012; Ogino *et al.*, 2008). Similarly, the presence of a functional LexA-binding site upstream of a XerD-type phage integrase suggests that the reported induction of integron integrases by the SOS response may also extend to some phage integrases (Guerin *et al.*, 2009). Finally, the putative SOS regulation of a death-on-curing protein is in agreement with and extends recent reports on the regulation of toxin-antitoxin systems by the SOS response in *E.coli*, which have important clinical implications due to their association with persister phenotypes (Dorr *et al.*, 2009; Singletary *et al.*, 2009; Wagner and Unoson, 2012).

## 4 CONCLUSIONS

In this work, we provide proof-of-concept that metagenomic data can be effectively leveraged to study TRNs by analyzing the SOS meta-regulon contributed by Gram-positive bacteria in the human gut microbiome. The use of metagenomic data in conjunction with a known conserved regulatory motif and *in vitro* techniques allows us to interrogate this regulatory network in its natural setting. Our analysis allows us to define the core components of the human gut SOS meta-regulon, revealing a substantial presence of error-prone polymerases, and identifies putative novel SOS members involved in control of cell division, virulence and mobile genetic element dissemination. The methodology introduced here is not constrained by regulon size or motif information content and can be applied to other metagenomic datasets and transcription factors meeting two basic criteria: a well-defined binding motif and experimental or *in silico* evidence of its conservation among the taxa of interest. This provides the means to exploit metagenomic data to enhance regulatory network inference.

*Funding*: UMBC Office of Research through a Special Research Assistantship/Initiative Support (SRAIS) award and by US National Science Foundation [MCB-1158056]. Fundació Cellex (fellowship to N.S.A.). The UMBC High Performance Computing Facility is supported by the US National Science Foundation MRI [CNS-0821258, CNS-1228778] and SCREMS [DMS-0821311] programs and by the University of Maryland, Baltimore County (UMBC).

*Conflict of Interest*: none declared.

## Supplementary Material

Supplementary Data
