# Supplementary material for: Characterization of the SOS meta-regulon in the human gut microbiome
Source: Bioinformatics. 2014 Jan 8;30(9):1193–7. doi: 10.1093/bioinformatics/btt753 (PMC3998124; doi:10.1093/bioinformatics/btt753)
Supplement: Supplementary Data [file supp_30_9_1193__index.html]

Characterization of the SOS meta-regulon in the human gut microbiome — Characterization of the SOS meta-regulon in the human gut microbiome — Characterization of the SOS meta-regulon in the human gut microbiome — Supplementary Data 

# Characterization of the SOS meta-regulon in the human gut microbiome

## Supplementary Data

files

**Files in this Data Supplement:**

- Supplementary Data - zip file
