# Supplementary material for: Characterization of the SOS meta-regulon in the human gut microbiome
Source: Bioinformatics. 2014 Jan 8;30(9):1193–7. doi: 10.1093/bioinformatics/btt753 (PMC3998124; doi:10.1093/bioinformatics/btt753)
Supplement: Supplementary Data [file supp_btt753_suppl_data.zip › Figure_S1.pdf]

Labelled *recA* promoter *B. subtilis*	+	+	+	+
LexA protein *B. subtilis*	-	+	+	+
Unlabelled *recA* promoter *B. subtilis*	-	-	+	-
Unlabelled *recA* promoter *E. coli*	-	-	-	+

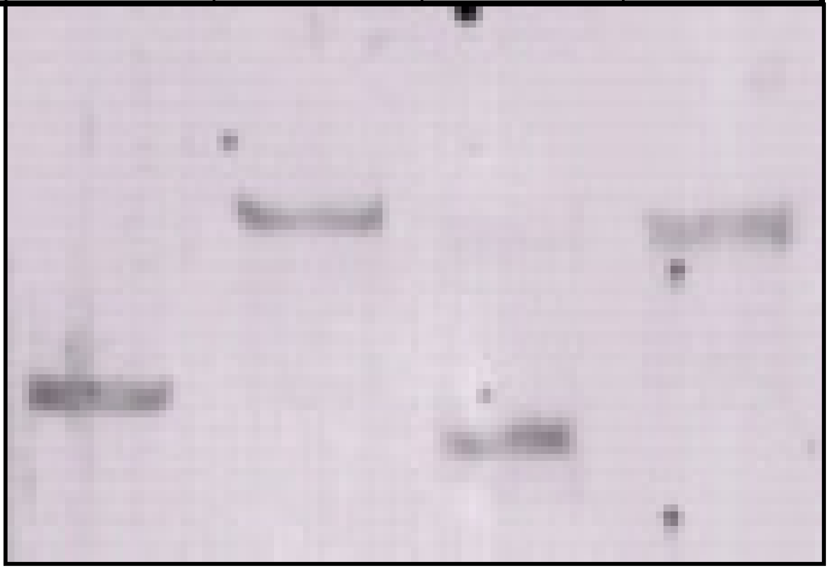

Figure S1 – EMSA specificity experiments (PDF). The lanes show, respectively, the standard EMSA using *B. subtilis* LexA and *recA* promoter, the competition assay adding 200-fold excess of unlabelled *recA* promoter, and the competition assay adding 200-fold excess of unlabelled *E. coli recA* promoter.
