# Supplementary material for: Characterization of the SOS meta-regulon in the human gut microbiome
Source: Bioinformatics. 2014 Jan 8;30(9):1193–7. doi: 10.1093/bioinformatics/btt753 (PMC3998124; doi:10.1093/bioinformatics/btt753)
Supplement: Supplementary Data [file supp_btt753_suppl_data.zip › Figure_S2.pdf]

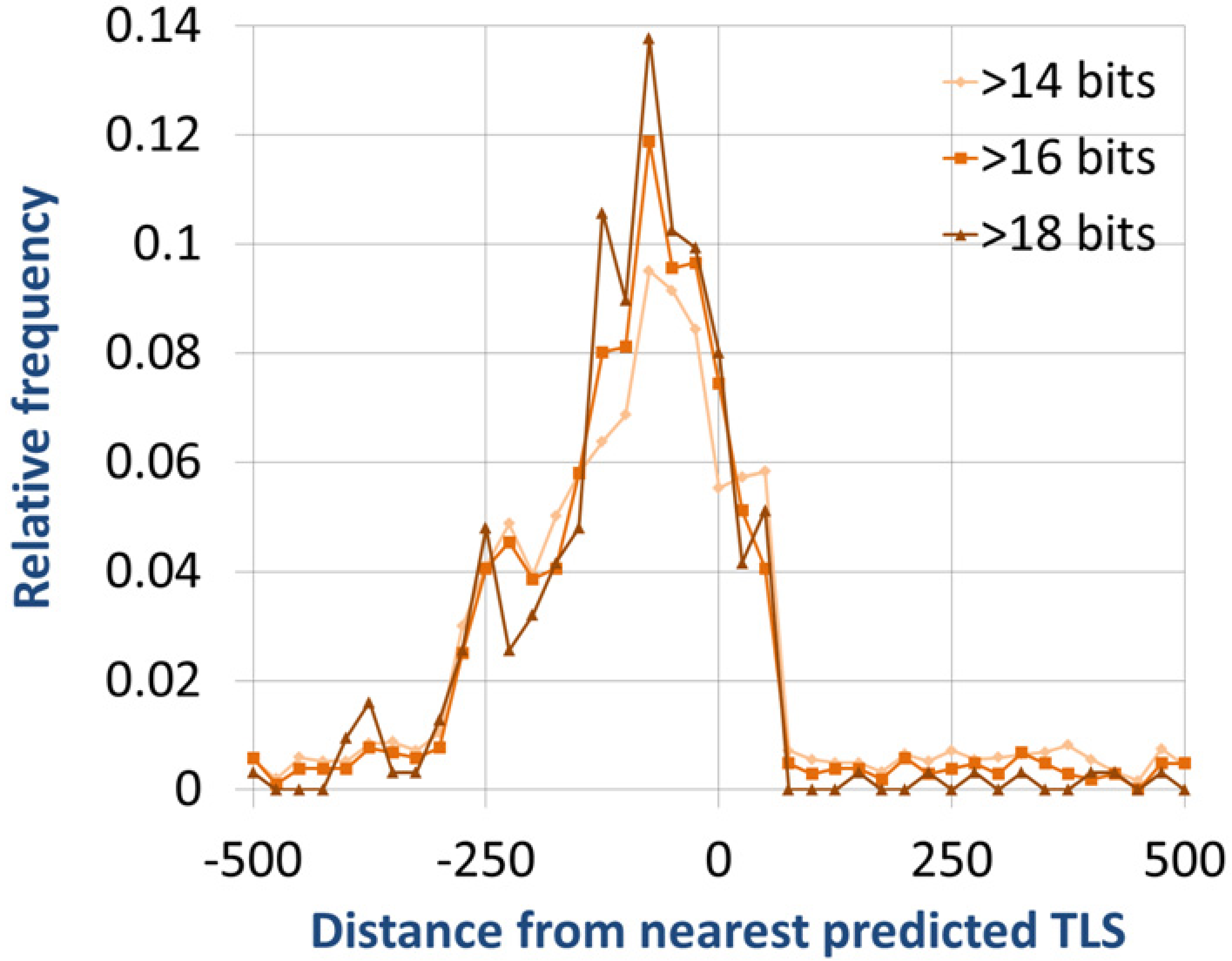

Figure S2 – Positional variation of LexA-binding site scores (PDF). Distribution of putative LexA-binding sites detected in the human gut microbiome data at different score thresholds (14, 16 and 18 bits) as a function of their distance to the closest predicted translational start site (TLS).
