# Supplementary material for: Characterization of the SOS meta-regulon in the human gut microbiome
Source: Bioinformatics. 2014 Jan 8;30(9):1193–7. doi: 10.1093/bioinformatics/btt753 (PMC3998124; doi:10.1093/bioinformatics/btt753)
Supplement: Supplementary Data [file supp_btt753_suppl_data.zip › Figure_S3.pdf]

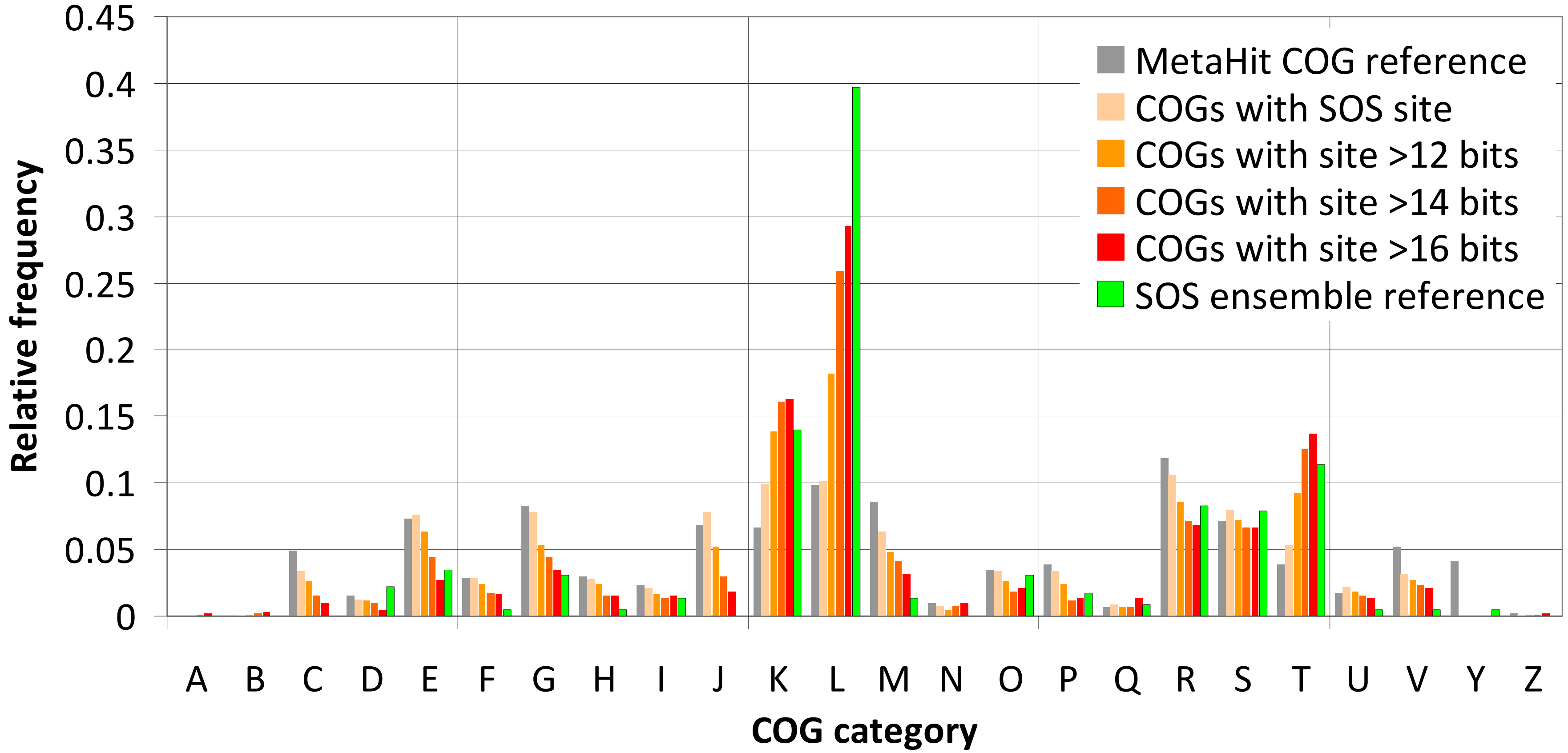

Figure S3 – Site dependent frequency of COG categories (PDF). Distribution of all COG categories as a function of site location and score threshold and comparison with a reference distribution of SOS response COG categories. COG category abbreviations are as follows: A - RNA processing and modification, B - Chromatin Structure and dynamics, C - Energy production and conversion, D - Cell cycle control and mitosis, E - Amino Acid metabolism and transport, F - Nucleotide metabolism and transport, G - Carbohydrate metabolism and transport, H - Coenzyme metabolis, I - Lipid metabolism, J - Translation, K - Transcription, L - Replication and repair, M - Cell wall/membrane/envelop biogenesis, N - Cell motility, O - Post-translational modification, protein turnover, chaperone functions, P - Inorganic ion transport and metabolism, Q - Secondary Structure, T - Signal Transduction, U - Intracellular trafficking and secretion, V - Defense mechanisms, Y - Nuclear structure, Z – Cytoskeleton, R - General Functional Prediction only, S - Function Unknown.
