# Supplementary material for: Characterization of the SOS meta-regulon in the human gut microbiome
Source: Bioinformatics. 2014 Jan 8;30(9):1193–7. doi: 10.1093/bioinformatics/btt753 (PMC3998124; doi:10.1093/bioinformatics/btt753)
Supplement: Supplementary Data [file supp_btt753_suppl_data.zip › Figure_S4.pdf]

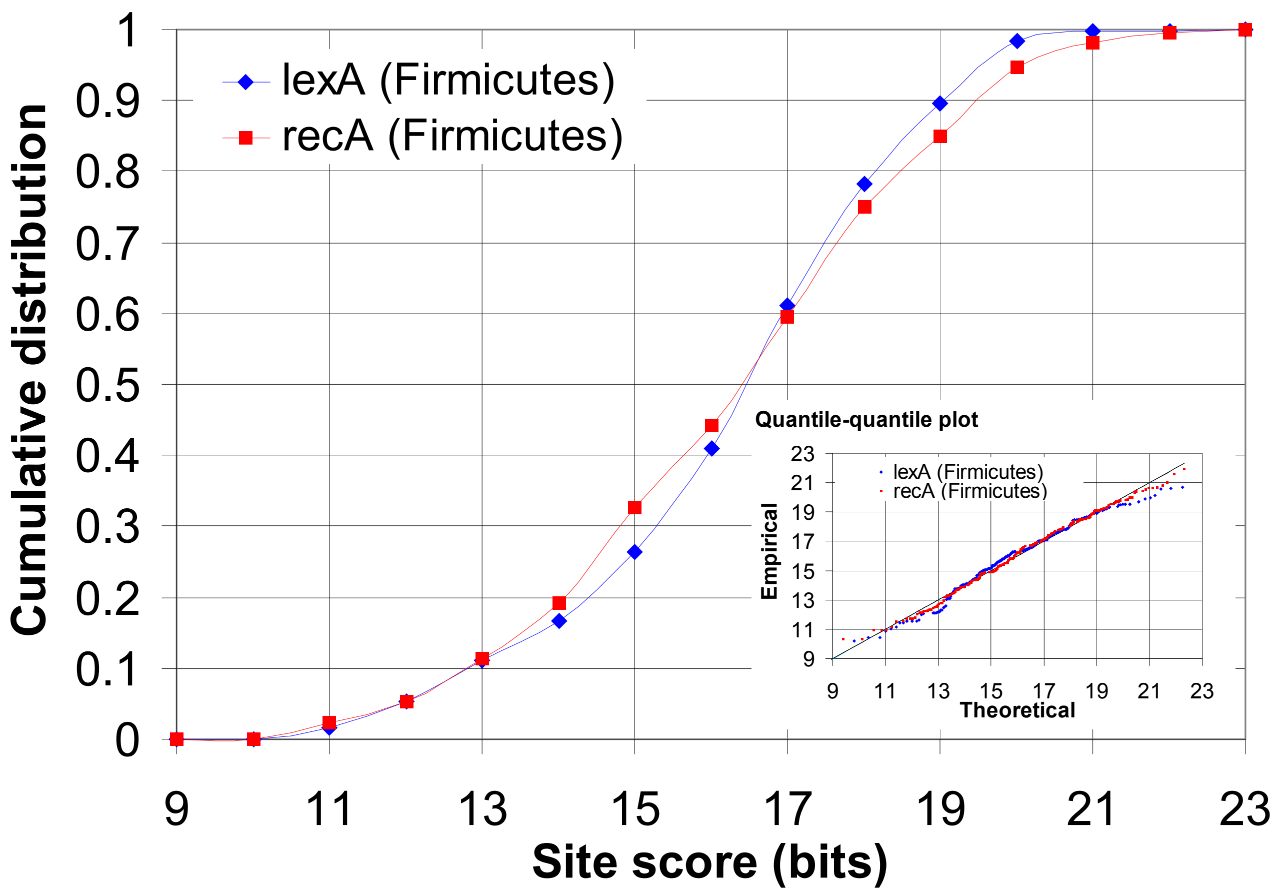

Figure S4 – Distribution of site scores in prototypical SOS genes (PDF). Cumulative distribution and quantile-quantile plot (inset) for putative LexA-binding sites upstream of *lexA* and *recA* genes from 308 Firmicutes genomes. Gene upstream regions for selected genes and genomes were downloaded using the Integrated Microbial Genomes (IMG) service of the Joint Genome Institute (JGI). Species were manually selected to represent without duplicates all Firmicutes genera with complete or draft genome sequences available. If multiple sites were present in a given promoter region, only the best-scoring site was used. A normal model (*lexA*: μ=16.2 , σ=2.3; *recA*: μ=16.3 , σ=2.5) for both distributions is not rejected under a Kolmogorov–Smirnov test (p >0.05).
