# Supplementary material for: Characterization of the SOS meta-regulon in the human gut microbiome
Source: Bioinformatics. 2014 Jan 8;30(9):1193–7. doi: 10.1093/bioinformatics/btt753 (PMC3998124; doi:10.1093/bioinformatics/btt753)
Supplement: Supplementary Data [file supp_btt753_suppl_data.zip › Figure_S5.pdf]

LexA (Gammaproteobacteria)

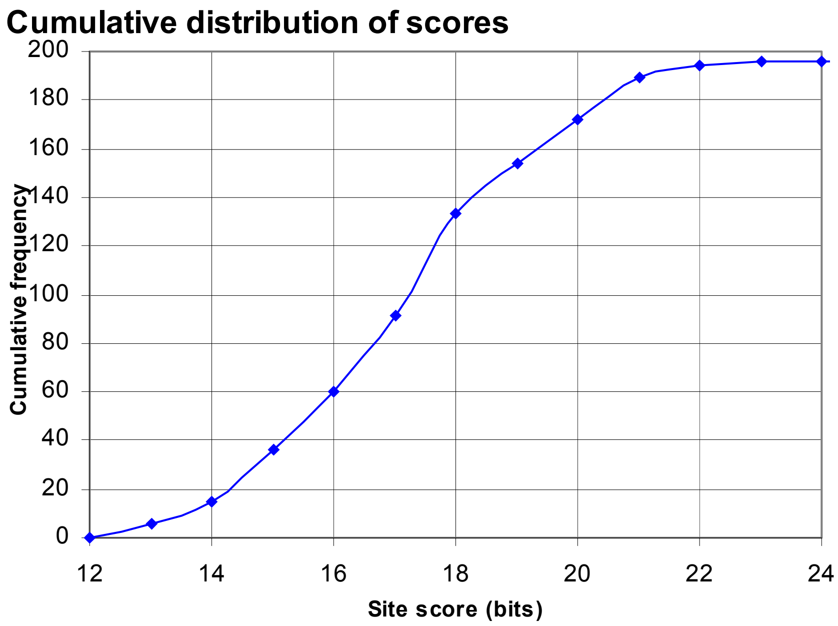

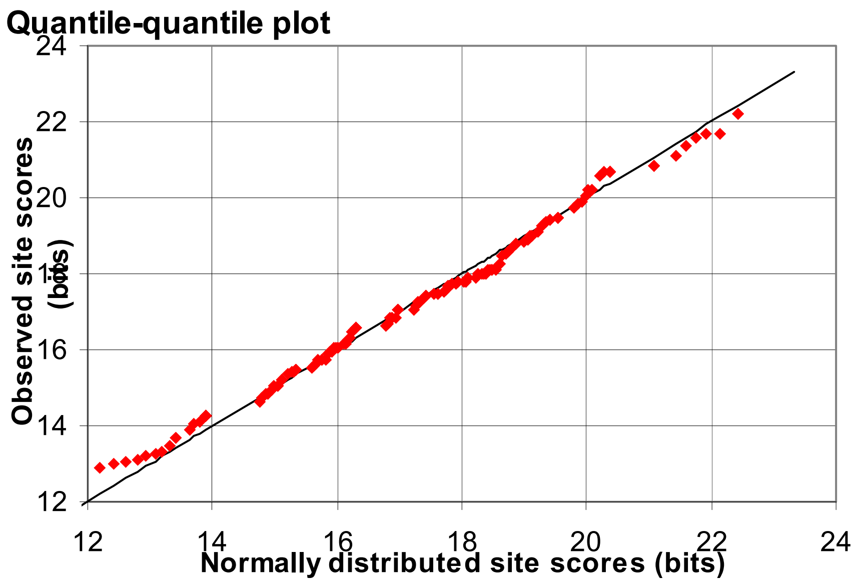

Figure S5 – Distribution of site scores in prototypical genes for other transcriptional regulators (PDF). Cumulative distribution and quantile-quantile plot (inset) for putative TF-binding sites upstream of genes coding for the transcription factor as conserved in 202 Gammaproteobacteria genomes, for the following transcription factors (LexA, Fur and CRP). TF-binding motifs were obtained from the Prodoric database. Gene upstream regions for selected genes and genomes were downloaded using the Integrated Microbial Genomes (IMG) service of the Joint Genome Institute (JGI). Species were manually selected to represent without duplicates all Firmicutes/Gammaproteobacteria genera with complete or draft genome sequences available. If multiple sites were present in a given promoter region, only the best-scoring site was used. A normal model for the observed distributions was not rejected under a Kolmogorov–Smirnov test ($p > 0.05$).

CRP (Gammaproteobacteria)

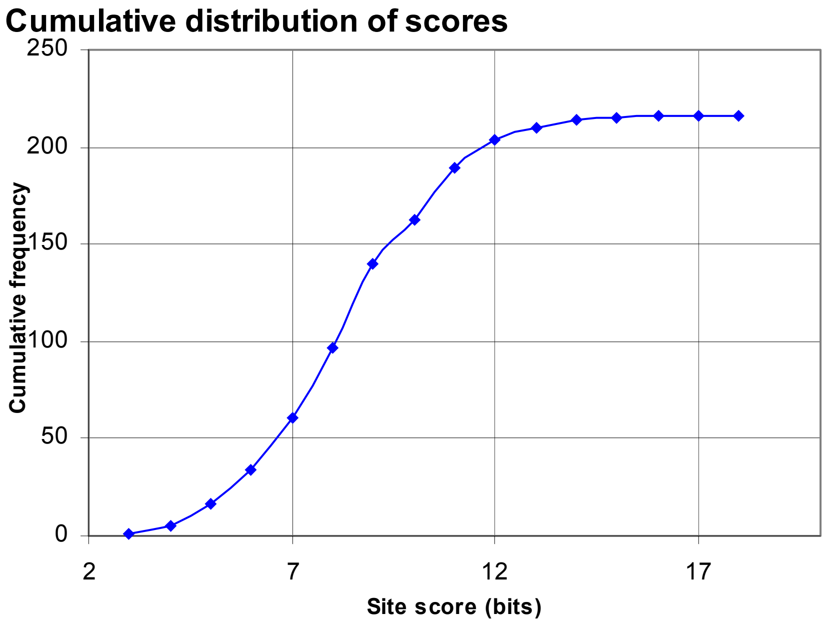

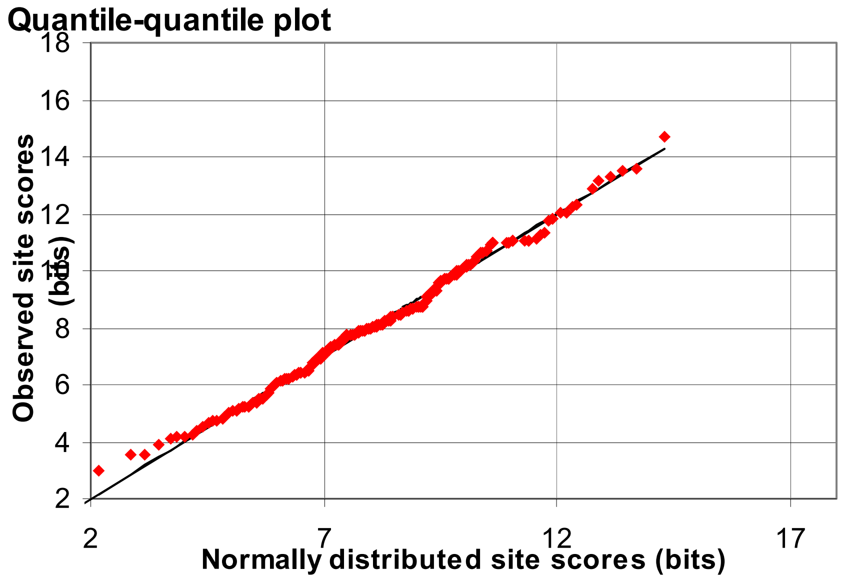

Figure S5 – Distribution of site scores in prototypical genes for other transcriptional regulators (PDF). Cumulative distribution and quantile-quantile plot (inset) for putative TF-binding sites upstream of genes coding for the transcription factor as conserved in 202 Gammaproteobacteria genomes, for the following transcription factors (LexA, Fur and CRP). TF-binding motifs were obtained from the Prodoric database. Gene upstream regions for selected genes and genomes were downloaded using the Integrated Microbial Genomes (IMG) service of the Joint Genome Institute (JGI). Species were manually selected to represent without duplicates all Firmicutes/Gammaproteobacteria genera with complete or draft genome sequences available. If multiple sites were present in a given promoter region, only the best-scoring site was used. A normal model for the observed distributions was not rejected under a Kolmogorov–Smirnov test ($p > 0.05$).

Fur (Gammaproteobacteria)

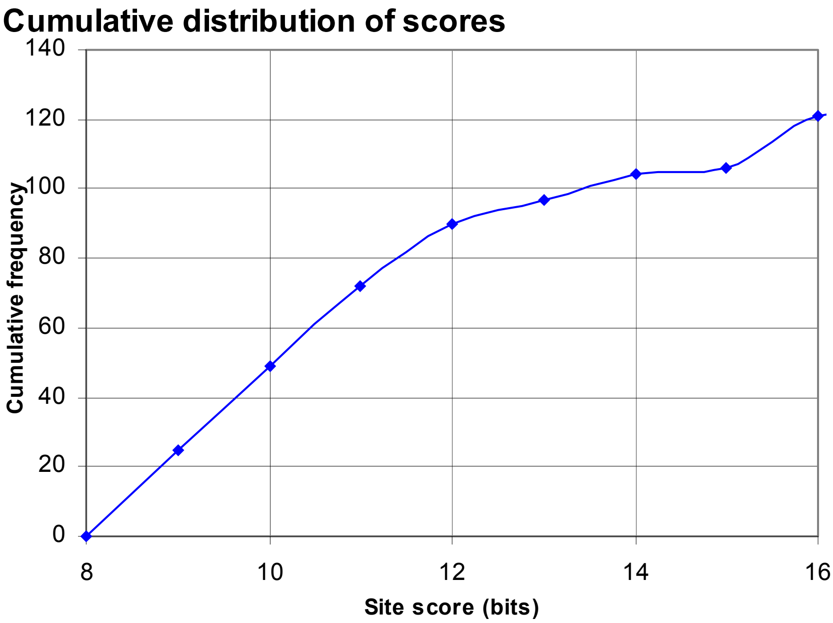

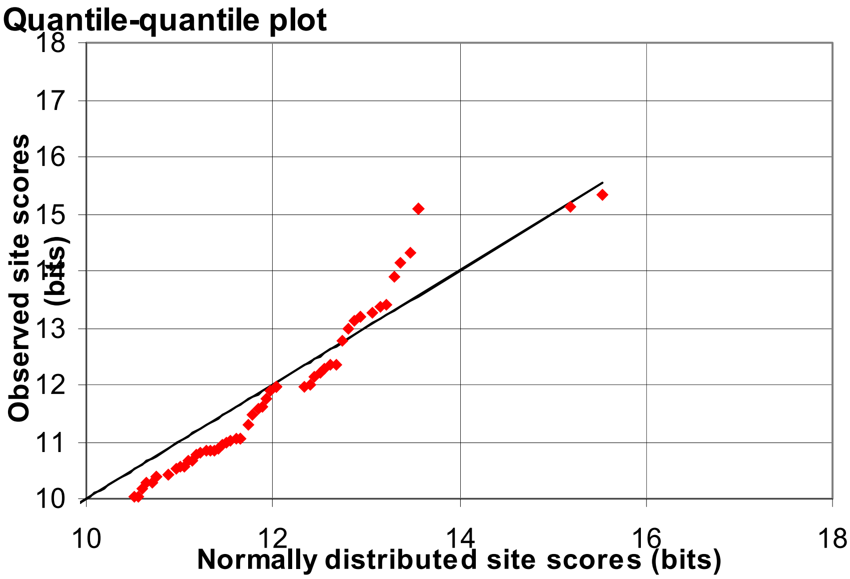

Figure S5 – Distribution of site scores in prototypical genes for other transcriptional regulators (PDF). Cumulative distribution and quantile-quantile plot (inset) for putative TF-binding sites upstream of genes coding for the transcription factor as conserved in 202 Gammaproteobacteria genomes, for the following transcription factors (LexA, Fur and CRP). TF-binding motifs were obtained from the Prodoric database. Gene upstream regions for selected genes and genomes were downloaded using the Integrated Microbial Genomes (IMG) service of the Joint Genome Institute (JGI). Species were manually selected to represent without duplicates all Firmicutes/Gammaproteobacteria genera with complete or draft genome sequences available. If multiple sites were present in a given promoter region, only the best-scoring site was used. A normal model for the observed distributions was not rejected under a Kolmogorov–Smirnov test ($p > 0.05$).
