# Supplementary material for: Characterization of the SOS meta-regulon in the human gut microbiome
Source: Bioinformatics. 2014 Jan 8;30(9):1193–7. doi: 10.1093/bioinformatics/btt753 (PMC3998124; doi:10.1093/bioinformatics/btt753)
Supplement: Supplementary Data [file supp_btt753_suppl_data.zip › Supplementary_material.docx]

Supplementary material

**Table S1 – Experimental LexA-binding sites (CSV).** Collection of experimentally-validated LexA-binding sites in Gram-positive bacteria used to generate the search motif shown in **Figure 1**A.

**Table S2 – Reference SOS regulons (CSV).** List of experimentally-reported SOS-regulated genes and associated COGs and COG categories in 19 different species.

**Table S3 – Detected LexA-binding sites (CSV).** List of putative Gram-positive LexA-binding sites identified in the MetaHit metagenome data within the promoter region of protein-coding genes taxonomically associated with Gram-positive bacteria. For each site, the sequence, score, position within the scaffold and all associated genes and COGs are reported.

**Table S4 – Experimental site information (CSV).** List of sites experimentally validated with EMSA. For each site, the site and EMSA oligonucleotide sequence, location in the metagenome and associated gene/COG information are reported.

**Table S5 – Cell Cycle Control (D) category SOS COGs (CSV).** List of experimentally-reported SOS-regulated genes mapping to COGs that belong to the Cell Cycle Control (D) category.

**Table S6 – Putative SOS COGs (CSV).** List of COGs putatively associated with the SOS response as a function of the linear regression coefficient of determination (*R^2^*) for the site score cumulative distribution in the 12-20 bit range. Only COGs with more than 10 sites in the 12-20 bit range and at least one site with score above 16 bits are reported.

**Figure S1 – EMSA specificity experiments (PDF).** The lanes show, respectively, the standard EMSA using *B. subtilis* LexA and *recA* promoter, the competition assay adding 200-fold excess of unlabelled *recA* promoter, and the competition assay adding 200-fold excess of unlabelled *E. coli* *recA* promoter.

**Figure S2 – Positional variation of LexA-binding site scores (PDF).** Distribution of putative LexA-binding sites detected in the human gut microbiome data at different score thresholds (14, 16 and 18 bits) as a function of their distance to the closest predicted translational start site (TLS).

**Figure S3 – Site dependent frequency of COG categories (PDF).** Distribution of all COG categories as a function of site location and score threshold and comparison with a reference distribution of SOS response COG categories. COG category abbreviations are as follows: A - RNA processing and modification, B - Chromatin Structure and dynamics, C - Energy production and conversion, D - Cell cycle control and mitosis, E - Amino Acid metabolism and transport, F - Nucleotide metabolism and transport, G - Carbohydrate metabolism and transport, H - Coenzyme metabolis, I - Lipid metabolism, J - Translation, K - Transcription, L - Replication and repair, M - Cell wall/membrane/envelop biogenesis, N - Cell motility, O - Post-translational modification, protein turnover, chaperone functions, P - Inorganic ion transport and metabolism, Q - Secondary Structure, T - Signal Transduction, U - Intracellular trafficking and secretion, V - Defense mechanisms, Y - Nuclear structure, Z – Cytoskeleton, R - General Functional Prediction only, S - Function Unknown.

**Figure S4 – Distribution of site scores in prototypical SOS genes (PDF**). Cumulative distribution and quantile-quantile plot (inset) for putative LexA-binding sites upstream of *lexA* and *recA* genes from 308 Firmicutes genomes. Gene upstream regions for selected genes and genomes were downloaded using the Integrated Microbial Genomes (IMG) service of the Joint Genome Institute (JGI). Species were manually selected to represent without duplicates all Firmicutes genera with complete or draft genome sequences available. If multiple sites were present in a given promoter region, only the best-scoring site was used. A normal model (*lexA*: *μ*=16.2 , *σ*=2.3; *recA*: *μ*=16.3 , *σ*=2.5) for both distributions is not rejected under a Kolmogorov–Smirnov test (p >0.05).

**Figure S5 – Distribution of site scores in prototypical genes for other transcriptional regulators (PDF**). Cumulative distribution and quantile-quantile plot (inset) for putative TF-binding sites upstream of genes coding for the transcription factor as conserved in 202 Gammaproteobacteria genomes, for the following transcription factors (LexA, Fur and CRP). TF-binding motifs were obtained from the Prodoric database. Gene upstream regions for selected genes and genomes were downloaded using the Integrated Microbial Genomes (IMG) service of the Joint Genome Institute (JGI). Species were manually selected to represent without duplicates all Firmicutes/Gammaproteobacteria genera with complete or draft genome sequences available. If multiple sites were present in a given promoter region, only the best-scoring site was used. A normal model for the observed distributions was not rejected under a Kolmogorov–Smirnov test (p >0.05).
